# 570. Impact of a Blood Culture Direct Identification Procedure on Time to Identification for Gram-Negative Organisms and Antimicrobial Management in Bloodstream Infections

**DOI:** 10.1093/ofid/ofad500.639

**Published:** 2023-11-27

**Authors:** Corwin Coppinger, Ashley L Cubillos

**Affiliations:** University of Colorado Skaggs School of Pharmacy and Pharmaceutical Sciences, Greeley, Colorado; Banner North Colorado Medical Center, Loveland, Colorado

## Abstract

**Background:**

Multiplex polymerase chain reaction (PCR) and microarray diagnostic methods for rapid identification (ID) in Gram-negative bloodstream infections (GNBSI) remain unavailable in many healthcare facilities. Use of other methods such as matrix-associated laser desorption/ionization time-of-flight (MALDI-TOF) along with rapid sample preparation from positive blood cultures, may decrease time to ID and targeted antimicrobial therapy in BSI. This study compares time to ID and final susceptibilities of Gram-negative bacteria (GNB) in positive blood cultures using a rapid MALDI-TOF direct ID process versus conventional culture methods, as well as impact on antimicrobial therapy in GNBSI.

**Methods:**

This retrospective cohort included patients ≥18 years of age who had a positive blood culture with a GNB from January 1 through December 31, 2022, at a hospital serviced by the microbiology lab at Banner University Medical Center Phoenix (conventional ID) or Banner University Medical Center Tucson (direct ID). Direct ID consisted of using MALDI-TOF for immediate ID of a centrifuged sample from a GNB-positive blood culture bottle. The primary outcome was time to GNB ID between the two groups. Secondary outcomes included time from positive blood culture to susceptibility reporting, definitive antibiotic regimen, and hospital discharge. Statistical analysis performed with Wilcoxon Rank-Sum testing for continuous data, and Chi square testing for nominal data.

**Results:**

A total of 254 patients were evaluated, with 100 included in each group. Median time to bacterial ID in the direct group vs. conventional group was 6.1 hr vs 29.2 hr (P< 0.0001). No differences in secondary outcomes, including times to final susceptibility (54.0 hr vs 60.9 hr, P=0.73), definitive antibiotic regimen (49.5 hr vs 44.9 hr, P=0.42), and hospital discharge (132.5 hr vs. 124.4 hr, P=0.85) were noted, though this study may have been unable to detect differences in such outcomes due to variance in organism resistance patterns and clinical practice between facilities.

**Conclusion:**

Direct MALDI-TOF ID significantly decreases time to organism identification in GNBSI compared with conventional methods. Utilization of direct ID plus antimicrobial stewardship interventions in GNBSI is an area of opportunity.

**Disclosures:**

**All Authors**: No reported disclosures

